# The Acute Phase Reactant Orosomucoid-1 Is a Bimodal Regulator of Angiogenesis with Time- and Context-Dependent Inhibitory and Stimulatory Properties

**DOI:** 10.1371/journal.pone.0041387

**Published:** 2012-08-14

**Authors:** Giovanni Ligresti, Alfred C. Aplin, Bruce E. Dunn, Ann Morishita, Roberto F. Nicosia

**Affiliations:** 1 Pathology and Laboratory Medicine Services, Veterans Administration Puget Sound Health Care System, Seattle, Washington, United States of America; 2 Department of Pathology, University of Washington, Seattle, Washington, United States of America; 3 Department of Pathology, Medical College of Wisconsin, Milwaukee, Wisconsin, United States of America; 4 Pathology and Laboratory Medicine Service, Department of Veteran Affairs Medical Center, Milwaukee, Wisconsin, United States of America; University of Washington, United States of America

## Abstract

**Background:**

Tissues respond to injury by releasing acute phase reaction (APR) proteins which regulate inflammation and angiogenesis. Among the genes upregulated in wounded tissues are tumor necrosis factor-alpha (TNFα) and the acute phase reactant orosomucoid-1 (ORM1). ORM1 has been shown to modulate the response of immune cells to TNFα, but its role on injury- and TNFα-induced angiogenesis has not been investigated. This study was designed to characterize the role of ORM1 in the angiogenic response to injury and TNFα.

**Methods and Results:**

Angiogenesis was studied with *in vitro*, *ex vivo*, and *in vivo* angiogenesis assays. Injured rat aortic rings cultured in collagen gels produced an angiogenic response driven by macrophage-derived TNFα. Microarray analysis and qRT-PCR showed that TNFα and ORM1 were upregulated prior to angiogenic sprouting. Exogenous ORM1 delayed the angiogenic response to injury and inhibited the proangiogenic effect of TNFα in cultures of aortic rings or isolated endothelial cells, but stimulated aortic angiogenesis over time while promoting VEGF production and activity. ORM1 inhibited injury- and TNFα-induced phosphorylation of MEK1/2 and p38 MAPK in aortic rings, but not of NFκB. This effect was injury/TNFα-specific since ORM1 did not inhibit VEGF-induced signaling, and cell-specific since ORM1 inhibited TNFα-induced phosphorylation of MEK1/2 and p38 MAPK in macrophages and endothelial cells, but not mural cells. Experiments with specific inhibitors demonstrated that the MEK/ERK pathway was required for angiogenesis. ORM1 inhibited angiogenesis in a subcutaneous *in vivo* assay of aortic ring-induced angiogenesis, but stimulated developmental angiogenesis in the chorioallantoic membrane (CAM) assay.

**Conclusion:**

ORM1 regulates injury-induced angiogenesis in a time- and context-dependent manner by sequentially dampening the initial TNFα-induced angiogenic response and promoting the downstream stimulation of the angiogenic process by VEGF. The context-dependent nature of ORM1 angioregulatory function is further demonstrated in the CAM assay where ORM1 stimulates developmental angiogenesis without exerting any inhibitory activity.

## Introduction

Tissues respond to injury, trauma or infection by swiftly releasing molecules that protect the host from invading organisms, prevent excessive cellular damage, promote the reparative process, and ultimately contribute to the restoration of normal function [1], [2]. This rapid reaction, known as the acute phase response (APR), is primarily mediated by macrophages which produce inflammatory cytokines when activated by microbial products or endogenous danger signals originating from dying cells [3], [4]. The innate capacity of the mononuclear phagocytic system to rapidly sense and react to noxious stimuli provides the host with a highly effective first line of defense prior to the full activation and implementation of adaptive immune responses.

During the APR inflammatory cytokines such tumor necrosis factor-α (TNFα), interleukin-1 (IL-1) and IL-6 stimulate the local and systemic production of a second wave of molecules known as acute phase proteins (APP) [1], [2]. Among the APP is orosomucoid-1 (ORM1), also known as α1-acid glycoprotein, a heavily glycosylated serum protein that has the capacity to bind and transport basic and neutral molecules. ORM1 is primarily synthesized by the liver but can be produced also in extrahepatic sites. Although its role in the APR remains unclear, ORM1 has been shown to have immunomodulatory and anti-inflammatory properties [5].

The immunosuppressive activity of APR is seemingly aimed at protecting the host against the detrimental side effects of an excessive inflammatory reaction. For example, ORM1 can inhibit neutrophil chemotaxis and superoxide production [6], [7], lymphocyte proliferation [8], and platelet aggregation [9], and can antagonize the capillary leakage caused by vascular permeability factors such as histamine and bradykinin [10]. ORM1 can also interfere with cytokine function by inducing the secretion of soluble TNFα receptor (sTNFR) and IL-1 receptor antagonist (IL1-Ra) [11]. When tested *in vivo*, ORM1 has the capacity to protect mice from lethal shock caused by TNFα and from inflammatory hepatitis induced by TNFα and galactosamine [12]. These protective properties are TNFα -specific since ORM1 has no effects against lipopolysaccharide (LPS)-induced shock [13] or anti-FAS induced hepatitis [14].

The immunomodulatory effects of ORM1 are, however, not exclusively anti-inflammatory. ORM1 has in fact the capacity to induce release of IL-1, TNFα, IL-6 and IL-12 by mononuclear leukocytes [5]. Since IL-1 and TNFα can in turn stimulate ORM1 production, ORM1 ultimately modulates the inflammatory reaction by contributing both anti- and pro-inflammatory signals to cytokine-mediated feedback mechanisms activated by the APR. Given the diverse functions of ORM1, contextual cues are likely to play a key role in the ultimate effects of this molecule on biologic processes triggered by the APR.

The mechanisms by which ORM1 mediates its functions are incompletely understood and not fully characterized. ORM1 has been shown to bind to the chemokine receptor CCR5 in macrophages [15] and the surface lectin-like receptor Siglec-5 (Sialic acid binding immunoglobulin-like lectin-5) in neutrophils [16]. ORM1 also binds to the asialoglycoprotein receptor in hepatocytes, but only after its sialic acid oligosaccharide chains are removed with neuraminidase [17]. Molecules implicated in ORM1 intracellular signaling include phospholipase-C, Src tyrosine kinases, and PI3 kinases [16], cAMP [18] and Rho/Rho kinases [16]. ORM1 can also modulate TNFα-induced phosphorylation of MAP kinases, c-Jun N-terminal kinase, and NFkB in macrophages [19].

Inflammatory cytokines produced during the APR have the capacity to promote angiogenesis, the process of formation of new blood vessels [20]. Angiogenic neovessels carry leukocytes, oxygen, and nutrients to the injured site and dispose of metabolic waste products, thereby contributing to defense mechanisms, reparative process and restoration of normal function. TNFα, a key cytokine produced by macrophages during the APR, induces the expression of angiogenic factors [21]–[24], promote formation of capillary tubes *in vitro* [25], [26], and stimulate angiogenesis *in vivo* [25], [27]–[29]. TNFα has also been shown to cause endothelial cytotoxicity [30], [31], inhibit endothelial cell proliferation [27], [30], [32] and suppress angiogenesis [31], [33]–[35]. The opposite effects of TNFα on angiogenesis have been variably attributed to contextual cues, TNFα doses, or activation of distinct TNFα signaling pathways [33], [36].

An additional regulatory mechanism of the angiogenic response to TNFα may involve molecules produced during the APR. Among these, ORM1 has recently been shown to enhance endothelial cell migration and capillary tube formation *in vitro* and angiogenesis in the chick chorioallantoic membrane (CAM) assay [37]. Since it is rapidly produced in response to TNFα, ORM1 can contextually modulate the angiogenic response to this cytokine in areas where ORM1-producing cells are most abundant [5]. Understanding how ORM1 regulates the angiogenic response to TNFα may have potential clinical implication since inflammatory angiogenesis contributes to the progression of many diseases including atherosclerosis, cancer, rheumatoid arthritis, and psoriasis [38].

In our laboratory we have studied the angiogenic response to injury by analyzing the *ex vivo* angioformative behavior of explants of rodent aortas. Aortic rings respond to the injury of the dissection procedure by producing vascular outgrowths that resemble vessels formed during angiogenesis *in vivo* [39], [40]. Using this model we found that aortic angiogenesis is dependent on adventitial macrophages which act as sensors of tissue injury and mediators of angiogenesis. We discovered that overexpression of TNFα by aortic macrophages plays a critical role in the cascade of gene activation that precedes angiogenic sprouting [41]. We also observed that aortic angiogenesis is preceded by overexpression by the aortic wall of ORM1 which is one of the most abundant proteins in the aortic conditioned medium [42], [43].

The present study was designed to investigate the role of ORM1 in the angiogenic response to injury. Our results indicate that ORM1, which is primarily expressed by mural cells, selectively inhibits injury- and TNFα-induced formation of microvessels during the early stages of the angiogenic response. The angiostatic activity of ORM1 is associated with inhibition of MAP kinases including MEK1/2 which is required for angiogenesis. This effect is TNFα-specific since ORM1 does not inhibit VEGF-mediated signaling and instead promotes VEGF production and angiogenesis over time. It is also cell-specific since ORM1 inhibits phosphorylation of MAP kinases in macrophages and endothelial cells but not mural cells. The context-dependent nature of the angiogenic modulatory activity of ORM1 is underscored by additional observations with *in vivo* models that ORM1 inhibits injury-induced angiogenesis but stimulates developmental angiogenesis. These results establish a novel mechanism of angiogenic regulation mediated by ORM1 during the acute phase response of the vessel wall to injury.

## Results

### ORM1 is upregulated during the early stages of aortic angiogenesis

Aortic rings cultured in collagen generate a self-limited angiogenic response that is triggered by the injury of the dissection procedure [39]. Angiogenesis in aortic cultures is mediated by endogenous mechanisms involving inflammatory cytokines and growth factors including TNFα and VEGF [41]. Among the molecules upregulated in aortic explants is the acute phase reactant ORM1 [42], [43]. ORM1 has the capacity to modulate the activity of inflammatory cytokines [12], [44], [45] but its role in the angiogenic response to injury has not been investigated.

**Figure 1:**
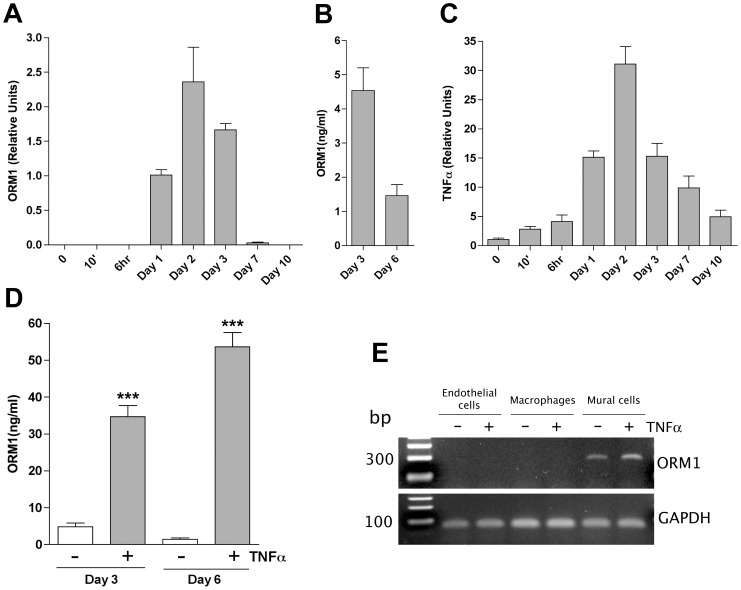
Expression of ORM1 in aortic ring cultures and isolated cells. (A). qRT-PCR shows that ORM1 is transiently expressed in aortic cultures during the early stages of angiogenesis (days 1–3). (B) ELISA of aortic culture conditioned media demonstrates highest production of ORM1 during the early stages of angiogenesis (day 1–3) compared to later stages (day 3–6). (C) TNFα is overexpressed rapidly (10 min) and before ORM1, peaks at day 2, and can be detected throughout the angiogenic response. (D). ELISA demonstrates marked stimulation of ORM1 production by TNFα during early and late stages of the angiogenic response (N = 3; *** = p<0.001). (E). PCR studies show that ORM1 is expressed in aortic mural cells but not in aortic endothelial cells or macrophages; ORM1 expression by mural cells is enhanced by TNFα.

To define the kinetics of ORM1 expression during angiogenesis, we performed qRT-PCR studies on aortic cultures at different stages of the angiogenic response. ORM1 expression started to increase at 24 hours, peaked at day 2–3, and rapidly decreased reaching barely detectable levels at day 7 (Fig. 1A). Changes in ORM1 mRNA correlated with ORM1 protein levels in the conditioned media (Fig. 1B) and overlapped with the early stages of angiogenic sprouting which became apparent at day 2–3 and peaked at day 7–8 [40].

### TNFα precedes and induces ORM1 gene expression during angiogenesis

We recently identified the macrophage-derived cytokine TNFα as a critical component of the cascade of gene activation responsible for the induction of angiogenesis in aortic cultures. Since TNFα is known to induce ORM1 expression [44], we performed additional qRT-PCR studies to determine the temporal relationship between TNFα and ORM1 expression. TNFα expression rapidly increased within minutes after injury, peaked at day 2 and decreased but remained relatively elevated following the angiogenic growth phase (Fig. 1C).

Taken together these studies indicate that ORM1 is downstream of TNFα in the cascade of gene activation associated with the angiogenic response to injury.

To evaluate the role of TNFα in ORM1 production we performed qRT-PCR and ELISA studies on TNFα-treated aortic ring cultures. TNFα markedly stimulated ORM1 production (Fig. 1D). ORM1 protein levels were notably high in control cultures and further increased from 4 ng/ml to 30–50 ng/ml in response to TNFα treatment (Fig. 1D).

### ORM1 is produced primarily by mural cells

Aortic outgrowths are composed of a mixed population of cells [43]. To determine the contribution of different cell types to ORM1 production, we analyzed ORM1 gene expression in isolated rat aortic macrophages, mural cells and endothelial cells. RT-PCR analysis showed that ORM1 was primarily expressed by mural cells. Interestingly, no significant expression of ORM1 was observed in endothelial cells or macrophages (Fig. 1E). TNFα treatment enhanced ORM1 expression in mural cells without noticeably affecting ORM1 expression in endothelial cells or macrophages. Thus mural cells are the primary source of ORM1 in aortic cultures.

### ORM1 has biphasic inhibitory and stimulatory effects at different stages of the angiogenic response

**Figure 2:**
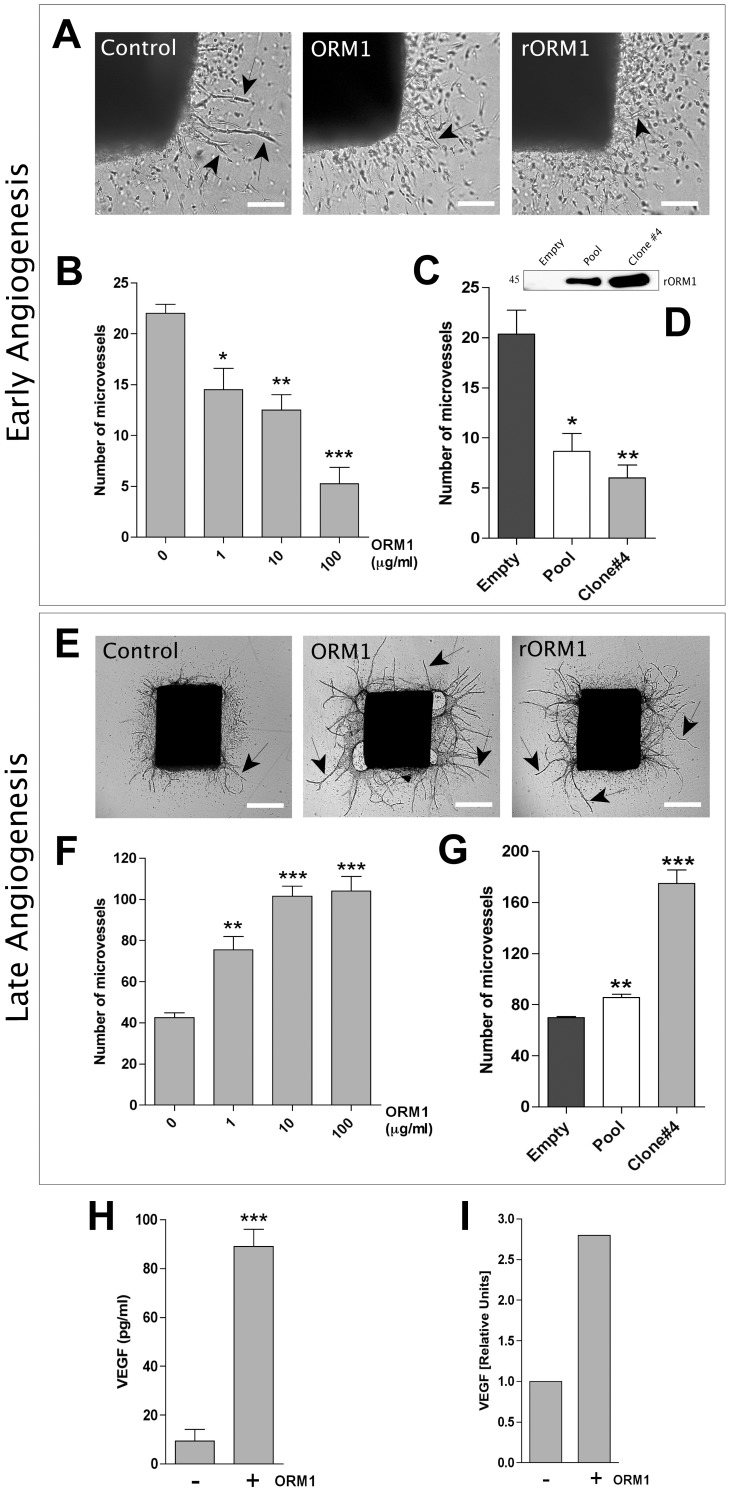
Effect of ORM1 on early and late stages of aortic angiogenesis. (A). Images of 3-day-old aortic ring cultures demonstrate reduced vessel sprouting (arrows) in cultures treated with serum-derived ORM1 or recombinant ORM1 (rORM1) compared to control. (B–C) The anti-angiogenic effect of both serum-derived (B) and recombinant (C) ORM1 in 3-day-old aortic ring cultures is dose dependent. (D). Western blot of rORM1 (conditioned media from EAhy926 cells clone # 4 and pooled clones) and control (EAhy926 cells transfected with empty vector) immunostained with anti-FLAG antibody. (E) Images of 9-day-old aortic ring cultures demonstrate stimulation of vessel sprouting by serum-derived ORM1 and rORM1 compared to control. (F–G) The pro-angiogenic effect of both serum-derived ORM1 (F) and rORM1 (G) in 9-day-old aortic ring cultures is dose dependent. (H). ELISA of conditioned media demonstrates marked stimulation of VEGF production by ORM1 in aortic cultures. (I). qRT-pCR shows induction of VEGF expression in aortic macrophages by ORM1. Magnification bars: 200 µm (A); 500 µm (E). * = p<0.05. ** = p<0.01; *** = p<0.001.

To characterize the effect of ORM1 on angiogenesis, collagen gel cultures of rat aorta were treated with increasing doses of this molecule. Aortic cultures treated with serum-derived ORM1 produced significantly fewer vessels than control during the initial phase of the angiogenic response (Fig. 2A, B). Maximum inhibitory effect was obtained with 100 µg/ml ORM1 which reduced endothelial sprouting to 10%–30% of control (Fig. 2B). The same inhibitory effect was observed in cultures treated with medium containing recombinant ORM1 (Fig. 2A, C, D). This anti-angiogenic effect was transient as continuous treatment with either serum-derived or recombinant ORM1 eventually stimulated angiogenesis causing a marked increase in vessel number and length (Fig. 2E–G). The proangiogenic effect of ORM1 was most pronounced at the concentration of 10–100 µg/ml. Stimulation of angiogenesis by ORM1 over time correlated with increased production of VEGF (Fig. 2H).

We previously showed that VEGF plays an important role in the endogenous mechanisms of injury-induced aortic angiogenesis [41], and found that macrophages of the aortic adventitia are required for optimal VEGF production and angiogenesis [41]. To evaluate the role of macrophages in ORM1-induced VEGF production, ORM1-treated macrophages were evaluated for VEGF expression. qRT-PCR studies showed that ORM1 induced overexpression of VEGF in macrophages (Fig. 2 I). ORM1-mediated upregulated expression of VEGF was most likely unrelated to hypoxia-induced mechanisms since experiments were conducted at atmospheric oxygen levels and qRT-PCR showed no effect of ORM1 on the expression of hypoxia inducible factor-1α (HIF1α), a known regulator of VEGF gene transcription (data not shown).

### ORM1 has opposite anti-angiogenic and pro-angiogenic effects on TNFα - and VEGF-treated cultures

**Figure 3:**
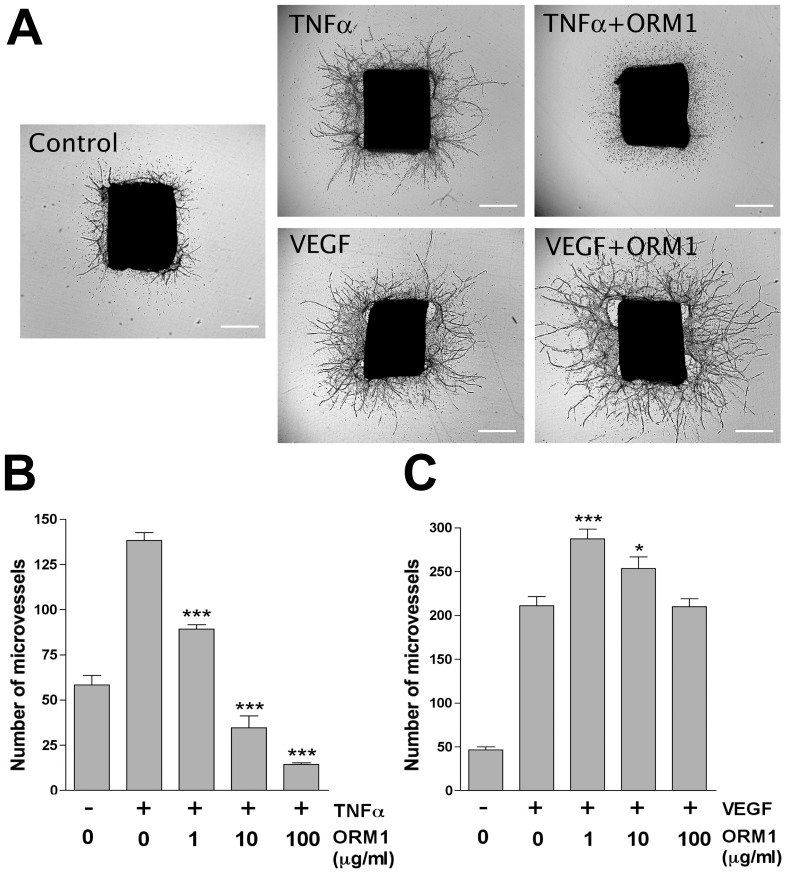
ORM1 differentially influences the effect of TNFα and VEGF on aortic angiogenesis. (A). Angiogenesis in aortic cultures is stimulated by both TNFα and VEGF (N = 4; p<0.001). TNFα (5 ng/ml) loses its pro-angiogenic activity and becomes inhibitory in the presence of ORM1 (100 µg/ml). Conversely ORM1 does not inhibit and instead enhances VEGF-stimulated angiogenesis (10 ng/ml). (B). TNFα progressively loses its capacity to stimulate angiogenesis in cultures treated with increasing doses of ORM1 and becomes potently inhibitory in the presence of 100 µg/ml ORM1. (C) Increasing doses of ORM1 have no inhibitory effect on the pro-angiogenic activity of VEGF which is actually enhanced by 1–10 µg/ml ORM1. Magnification bars: 500 µm (A). N = 4; * = p<0.05; *** = p<0.001.

Based on our previous observation that TNFα is critically involved in the early mechanisms of angiogenic induction and precedes VEGF overexpression [41], we hypothesized that the proangiogenic effect of this cytokine might be influenced by ORM1 which has been shown to modulate TNFα inflammatory activity [11], [12]. To investigate this possibility we treated aortic cultures with TNFα in the presence or absence of ORM1. TNFα -treated aortic ring cultures produced significantly more vessels than untreated controls as reported [41]. Concurrent treatment with ORM1 and TNFα completely abrogated the TNFα stimulatory effect on aortic angiogenesis (Fig. 3). This effect was TNFα -specific since ORM1 had no inhibitory activity on VEGF-stimulated cultures. ORM1 actually potentiated the angiogenic effect of VEGF, and cultures treated with both ORM1 and VEGF produced an even greater number of vessels than cultures treated with VEGF alone (Fig. 3).

### Priming of aorta with ORM1 impairs the angiogenic response to injury and the proangiogenic activity of TNFα

**Figure 4:**
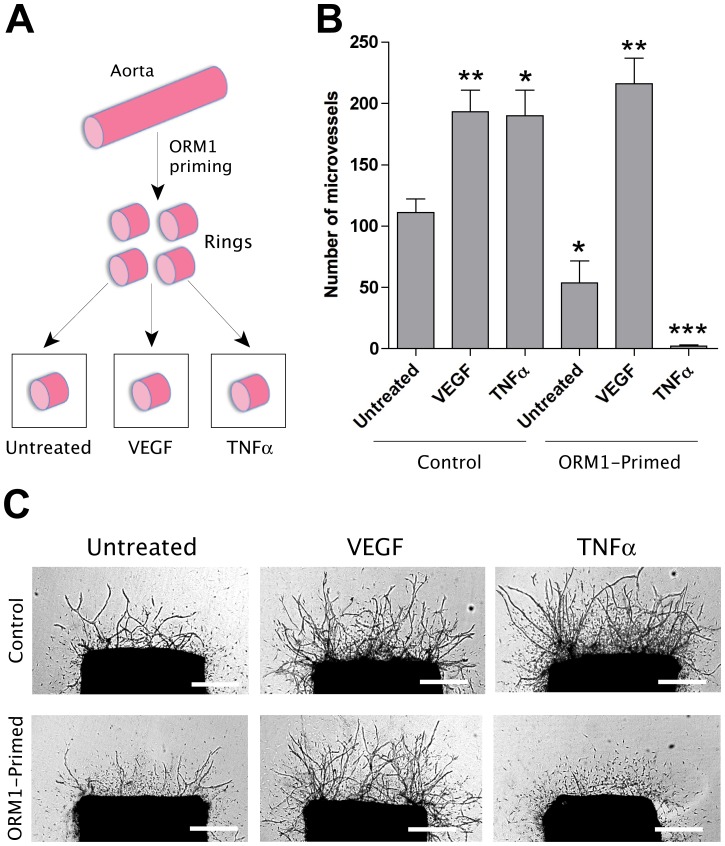
The angiogenic response of rings prepared from ORM1-primed aorta is markedly inhibited by TNFα but not VEGF. (A) Schematic drawing showing rings prepared from aortic tubes primed with 100 µg/ml ORM1 and then cultured in collagen gels with or without VEGF (10 ng/ml) or TNFα (10 ng/ml). (B–C). VEGF stimulates both control and ORM1-primed aortic rings whereas TNFα stimulates control rings but potently inhibits the angiogenic response of ORM1-primed rings. Magnification bars: 500 µm (C). N = 4; * = p<0.05. ** = p<0.01; *** = p<0.001.

The transient anti-angiogenic effect of ORM1 and its capacity to suppress the proangiogenic activity of TNFα suggested that this molecule might be able to inhibit angiogenic mechanisms operating prior to endothelial sprouting in response to injury. To investigate this hypothesis we incubated whole aortic tubes overnight in ORM1-containing serum-free medium prior to preparation of the aortic rings, i.e. before injuring the aorta with a scalpel blade. Rings obtained from either aortic tubes pretreated with ORM1 or untreated control tubes were cultured in collagen gels under serum-free conditions and in the absence of ORM1. Rings prepared from ORM1-primed aortic tubes produced significantly fewer vessels than control rings (Fig. 4). At variance with the transient inhibitory effect observed in cultures continuously treated with ORM1, the inhibitory effect obtained with the ORM1 pretreatment method persisted for the duration of the experiment and showed no evidence of the delayed stimulatory effect observed in cultures continuously treated with exogenous ORM1. This indicated that ORM1 had the capacity to selectively impair endogenous pro-angiogenic mechanisms triggered by injury, provided that the aortic wall had been primed with this molecule prior to the preparation of the rings.

To further evaluate the effect of ORM1 on angiogenesis, aortic tubes were primed with ORM1 and then cultured in collagen gels with or without TNFα or VEGF. The angiogenic response was completely suppressed in cultures of ORM1-primed aortas treated with TNFα (Fig. 4). This inhibitory effect was not due to toxicity since endothelial cells sprouted upon withdrawal of exogenous TNFα (data not shown). The response of the aortic rings to VEGF was instead unaffected by ORM1 priming (Fig. 4).

### Priming with ORM1 inhibits TNFα- and macrophage-induced sprouting of endothelial cells in a collagen gel invasion assay

**Figure 5:**
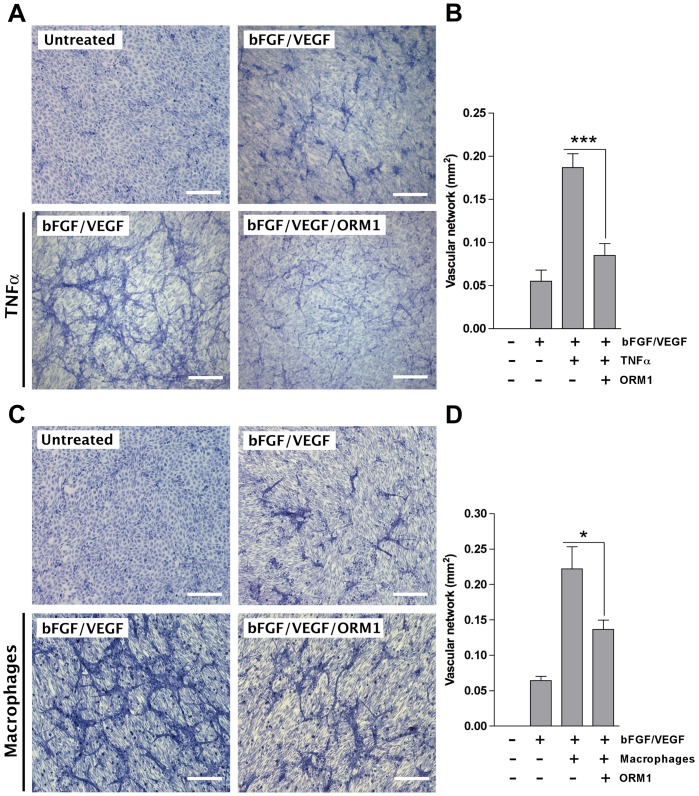
ORM1-priming impairs the capacity of TNFα or macrophages to promote angiogenic sprouting in a collagen invasion assay with isolated endothelial cells. (A–B). TNFα (10 ng/ml) markedly enhances formation of capillary tubes in response to bFGF/VEGF (50 ng/ml each); priming of endothelial cells with ORM1 (100 µg/ml) completely abrogates the TNFα stimulatory effect. (C–D) Macrophages embedded in the collagen gel enhance formation of capillary tubes in response to bFGF/VEGF; priming of the endothelial/macrophage co-culture with ORM1 significantly impairs the macrophage stimulatory effect. Magnification bars = 200 µm. * = p<0.05; *** = p<0.001.

To further evaluate the capacity of ORM1 to function as an inhibitor of TNFα proangiogenic activity, we tested the ORM1 effect on the sprouting behavior of isolated endothelial cells in a collagen gel invasion assay. In this model endothelial cells invade collagen forming branching tubes in response to stimulation by exogenous angiogenic factors. Treatment with the bFGF/VEGF combination induces limited sprouting after 2 days (Fig. 5). Addition of TNFα markedly stimulated collagen invasion by sprouting endothelial cells leading to the rapid formation of extensive networks of branching endothelial tubes (Fig. 5A, B). Marked stimulation of angiogenesis was also obtained by adding macrophages to the collagen gel (Fig. 5C, D). Pretreatment with ORM1 rendered the endothelial cells refractory to the pro-angiogenic activity of TNFα or macrophages (Fig. 5 A–D). In contrast ORM1 did not interfere with bFGF/VEGF-induced endothelial sprouting (data not shown). These findings confirm in a model with isolated endothelial cells that ORM1 has the capacity to selectively block the angiogenic activity of TNFα.

### ORM1 attenuates injury- and TNFα-induced phosphorylation of MEK1/2 and p38 MAP kinase in aortic explants, but has no inhibitory effect on VEGF signaling

**Figure 6:**
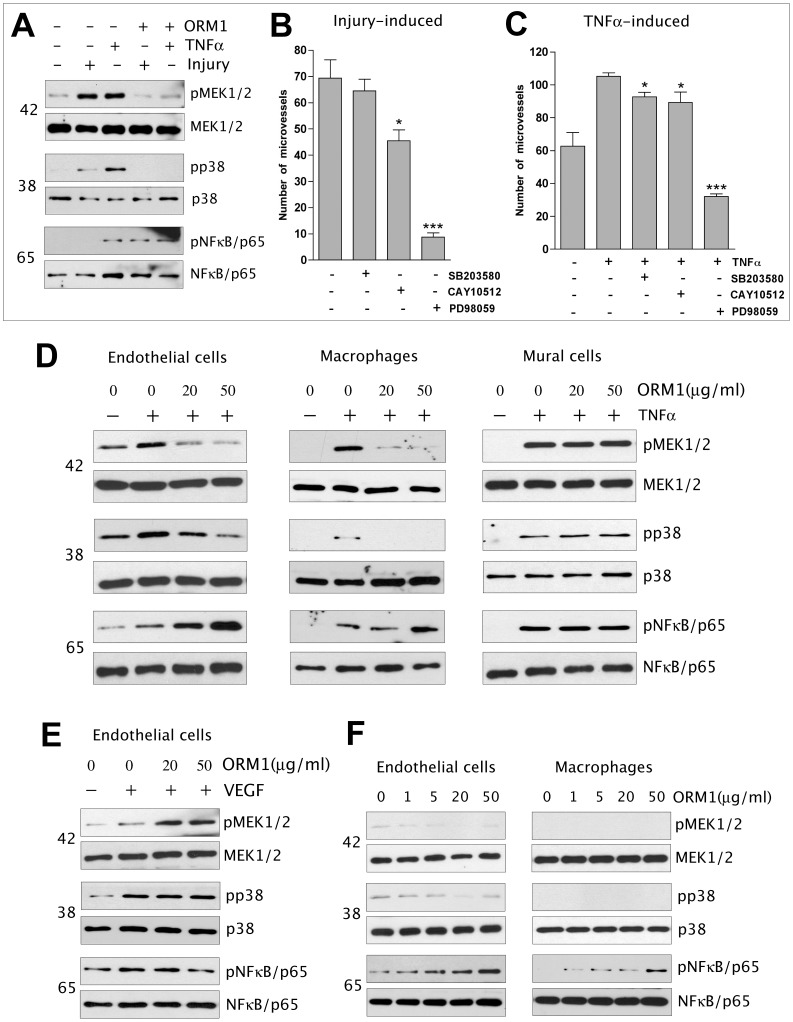
Effect of ORM1 on signal transduction in aortic explants and isolated endothelial cells, macrophages or mural cells. (A). ORM1 inhibits injury- and TNFα (20 ng/ml)-induced phosphorylation of MEK1/2 and p38, but has no effect on TNFα -induced phosphorylation of NFκB. (B–C) Injury-induced (B) and TNFα-induced (C) aortic angiogenesis are suppressed by the MEK1/2 inhibitor PD98059 (N = 4; *** = p<0.001) whereas inhibitors of other signaling pathways have no effect or are minimally inhibitory (* = p<0.05). (D). ORM1 inhibits TNFα-induced phosphorylation of MEK1/2 and p38, but has no inhibitory effect on TNFα -induced phosphorylation of NFκB in endothelial cells and macrophages; ORM1 has no effect on TNFα signaling in mural cells. (E). ORM1 has no inhibitory effect on VEGF (10 ng/ml) signaling in endothelial cells. (F). ORM1 dose dependently induces phosphorylation of NFκB in endothelial cells and macrophages, but has no significant effect on MEK1/2 and p38 phosphorylation.

TNFα effects are mediated by a complex network of signaling molecules including MEK1/2, p38 and NFkB. These pathways are also activated in response to injury [46]–[48]. To evaluate the effect of ORM1 on signal transduction events occurring in the aortic wall prior to angiogenesis, protein extracts from aortic tubes incubated overnight in serum-free medium with or without ORM1 and then injured by cross sectioning to prepare rings or treated with TNFα without being cross sectioned were evaluated by Western analysis for changes in phosphorylation of signaling molecules. Rings examined 30 min after injuring the aorta exhibited phosphorylation of MEK1/2 and p38. Treatment of aortic tubes with TNFα for 15 min induced phosphorylation of MEK1/2, p38 and NFkB. Pretreatment of the aortic tubes with ORM1 abrogated injury- and TNFα-induced phosphorylation of MEK1/2 and p38 but not of NFkB which was actually hyperphosphorylated (Fig. 6A).

To identify the cellular mediators of the ORM1 effect, further signaling studies were performed on isolated cells. Priming of endothelial cells or macrophages for 3 hours with ORM1 markedly attenuated TNFα-induced phosphorylation of MEK1/2 and p38 but had no inhibitory effect on NFkB which was instead hyperphosphorylated by ORM1; this effect was particularly pronounced in macrophages. Interestingly ORM1 had no effect on TNFα-induced phosphorylation of MEK1/2, p38 and NFkB in mural cells (Fig. 6D). In addition ORM1 did not inhibit VEGF-induced phosphorylation of MAP kinases in endothelial cells (Fig. 6E). Endothelial cells and macrophages treated with ORM1 for 30 min in the absence of TNFα or VEGF exhibited increased phosphorylation of NFkB but showed no significant changes in the phosphorylation of MEK1/2 or p38 (Fig. 6F). Taken together these results indicate that the capacity of ORM1 to inhibit TNFα signaling is cell- and signaling pathway-specific. They also demonstrate that ORM1 selectively interferes with TNFα signaling without affecting the VEGF signaling pathway.

### MEK/ERK signal transduction pathway is essential for the angiogenic response of the aorta to injury and to TNFα-stimulated aortic angiogenesis

To evaluate the contribution of signal transduction pathways modulated by ORM1 in the angiogenic response of the aorta to injury and exogenous TNFα or VEGF, collagen cultures were treated with cell signaling inhibitors. Treatment with the MEK1/2 inhibitor PD98059 resulted in a marked inhibition of the angiogenic response of the aortic wall to injury and exogenous TNFα. Aortic angiogenesis in control (injury-induced) and TNFα-treated cultures was minimally blocked by the NFkB inhibitor CAY50512 and unaffected by the p38 inhibitor SB203580 (Fig. 6B, C). These findings indicate that the MEK1/2 signaling pathway is essential for both injury- and TNFα-induced angiogenesis and implicate MEK1/2 dephosphorylation as a potential mechanism by which ORM1 suppresses TNFα-induced angiogenesis.

### ORM1 inhibits inflammatory angiogenesis in the *in vivo* aortic ring model and stimulates developmental angiogenesis in the chorioallantoic membrane

**Figure 7:**
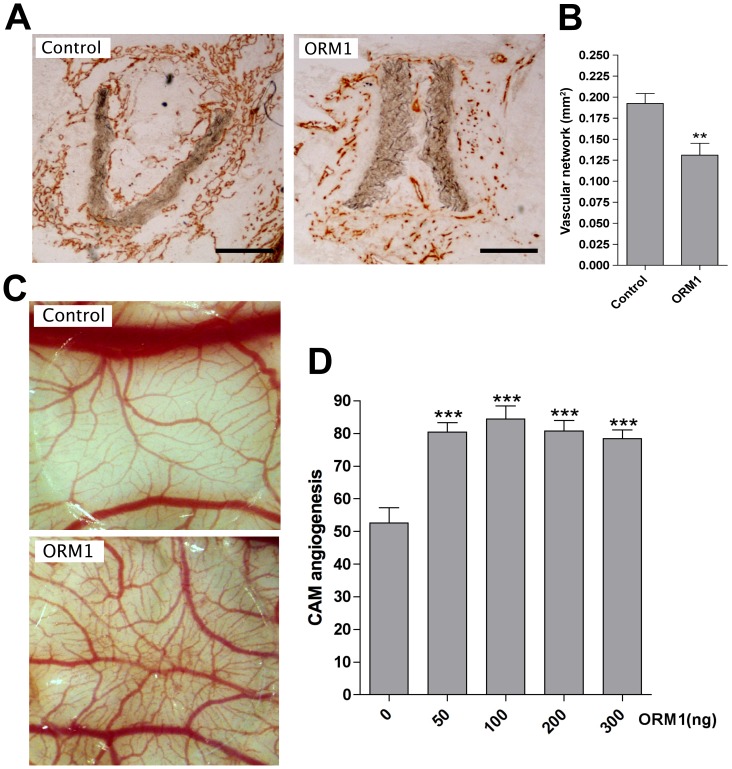
Effect of ORM1 on angiogenesis in the *in vivo* aortic ring and CAM assays. (A). Frozen sections of subcutaneously implanted aortic rings stained by immunoperoxidase for CD31 show fewer microvessels in ORM1-primed implants compared to control. (B). Quantitative analysis shows 32% reduction in angiogenesis in ORM1-primed implants (N = 12; ** = p<0.01). (C). Representative image of ORM1-treated CAM shows increased angiogenesis compared to control. (D). Quantitative analysis demonstrates 30% increase in blood vessels in CAMs treated with 50–300 ng ORM1 (N = 15; *** = p<0.001).

To evaluate the effect of ORM1 on *in vivo* angiogenesis we tested the effects of this molecule in the *in vivo* aortic ring and CAM assays. Angiogenesis in the *in vivo* aortic ring assay is induced by angiogenic factors and inflammatory cytokines/chemokines released by the aortic implant in the collagen gel in which it is embedded. The gel of control constructs without aortic rings has no angiogenic vessels [49]. Untreated aortic rings generated a prominent angiogenic response resulting in the invasion of the collagen gel by numerous CD31+ neovessels and the neovascularization of the aortic lumen. Angiogenesis was also observed in ORM1-soaked constructs containing ORM1-primed aortic rings, but the extent of neovessel formation in this group was significantly reduced (Fig. 7A, B).

The effect of ORM1 on developmental angiogenesis was studied in the CAM assay. The CAM of 10-day-old chick embryos was treated with increasing doses of ORM1 and analyzed 3 days later for extent of neovascularization. ORM1 in this system significantly stimulated angiogenesis (Fig. 7C, D).

These *in vivo* results indicate that ORM1 has context-dependent effects on the angiogenic response, and corroborate our *ex vivo*/*in vitro* observations that ORM1 inhibits the angiogenic response to injury and TNFα but has the capacity to promote VEGF-stimulated angiogenesis.

## Discussion

Orosomucoid-1 (ORM1) is a heavily glycosylated molecule characteristically overexpressed during the acute phase response to stressor signals such as infection, injury and inflammation [44]. Although its role in reactive and pathologic processes is not fully understood, ORM1 has been shown to have a number of regulatory functions including modulation of the immune response [5]. ORM1 is mainly produced by liver parenchymal cells, but can also be expressed at extra-hepatic sites [45]. We recently identified ORM1 transcripts among the genes overexpressed in angiogenic cultures of rat aorta [42]. Angiogenesis in the *ex vivo* aortic ring model is triggered by injury and preceded by overexpression of proangiogenic inflammatory cytokines including TNFα that are typical of the acute phase response [41]. Although ORM1 has been shown to regulate the activity of TNFα and other cytokines [11], there are no data on its capacity to influence cytokine-mediated angiogenesis. The present study was designed to fill this gap and better define the role of ORM1 in the angiogenic process.

The results of our study can be summarized as follows: (1) Aortic injury induces expression of TNFα and ORM1. (2) TNFα, which is produced by injury-activated macrophages [41], is overexpressed several hours before ORM1 transcripts become detectable. (3) TNFα induces ORM1 expression in aortic explants and isolated mural cells which are the primary source of ORM1. (4) ORM1 inhibits early aortic sprouting but stimulates VEGF production and angiogenesis over time. (5) ORM1 abrogates the pro-angiogenic effect of TNFα but does not inhibit and instead enhances the angiogenic activity of VEGF. (6) ORM1-primed aortic rings have a reduced angiogenic response to injury and are unable to sprout when treated with TNFα but are fully responsive to VEGF. (7) ORM1 inhibits TNFα-induced sprouting of isolated endothelial cells but has no effect on bFGF/VEGF-induced sprouting. (8) ORM1 attenuates injury- and TNFα-induced phosphorylation of MEK1/2 and p38 MAPK in aortic explants without affecting the capacity of TNFα to phosphorylate NFkB. (9) ORM1 inhibits TNFα-induced phosphorylation of MEK1/2 and p38 MAPK in endothelial cells and macrophages but has no effect on the phosphorylation of these MAP kinases in mural cells. (10) ORM1 has no inhibitory effect on VEGF-induced phosphorylation of MEK1/2 and p38 MAPK in aortic rings or isolated endothelial cells. (11) Pharmacologic inhibition of MEK1/2 abrogates the pro-angiogenic activity of TNFα- and inhibits injury-induced aortic angiogenesis. (12) Aortic rings obtained from ORM1-treated aorta have reduced angiogenic properties in a subcutaneous model of inflammatory angiogenesis. (13) Conversely, ORM1 stimulates developmental angiogenesis in the CAM assay.

These findings provide new insights into the mechanisms that govern the early stages of the angiogenic response to injury and identify ORM1 as one of its paracrine regulators. We first discovered that ORM1 has time- and context-dependent effects of on angiogenesis when we were testing its activity in the *ex vivo* aortic ring model of angiogenesis. This assay reproduces the angiogenic response in a chemically defined culture environment that can be easily monitored and quantified [39], [40]. Aortic angiogenesis is triggered by the injury of the aortic ring preparation procedure and mediated by endogenous inflammatory cytokines, chemokines and growth factors [42], [50] [43]. Among the endogenous factors produced by the aorta is TNFα, which is produced by activated resident macrophages within minutes after injury. TNFα is one the mediators of aortic injury-induced VEGF expression, and TNFα gene disruption causes marked reduction in VEGF mRNA/protein levels and angiogenesis [41].

**Figure 8:**
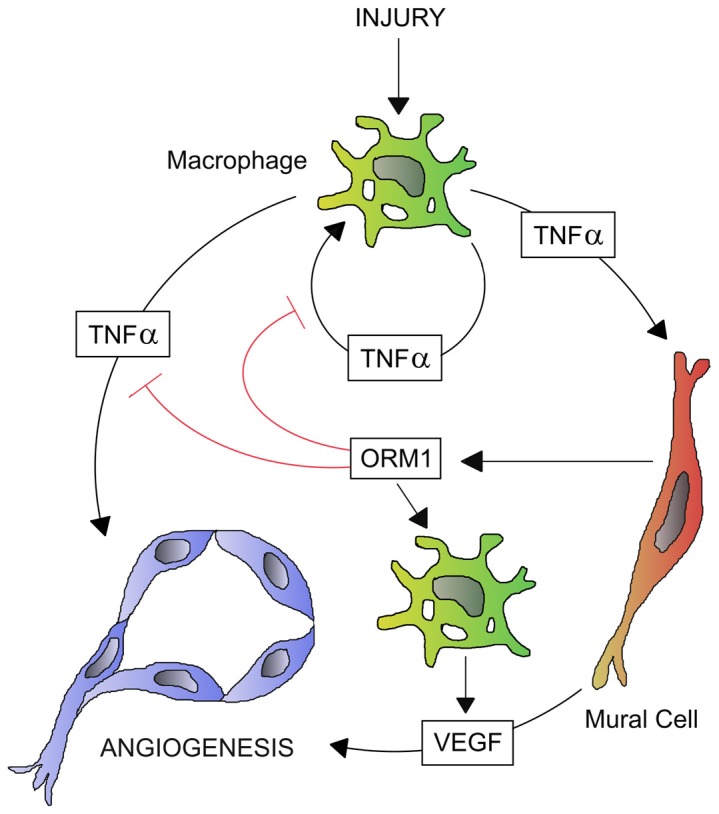
Schematic drawing showing regulation of injury-induced angiogenesis by ORM1. Macrophages produce TNFα in response to injury. TNFα stimulates angiogenesis directly by activating endothelial cells and indirectly by inducing production of VEGF by mural cells. TNFα also induces production of ORM1 by mural cells. ORM1 in turn suppresses TNFα signaling in macrophages and endothelial cells but has no inhibitory effect on mural cell signaling. ORM1 induces VEGF production by macrophages and promotes VEGF-mediated angiogenesis over time. Thus ORM1 fine tunes the angiogenic response by dampening TNFα stimulatory signals and by promoting VEGF-mediated downstream pro-angiogenic effects.

The angiogenic response of the aortic wall is limited in time as vessels start growing 2–3 days after the initial injury and stop growing at day 7–8. In order for this process to be self-limited, negative feedback mechanisms must be activated to prevent unrestrained vascular proliferation. To that end ORM1, produced in response to TNFα, blocks the TNFα-mediated pathway of angiogenic induction once downstream proangiogenic signals under the control of VEGF have been fully activated. ORM1 accomplishes this task by inhibiting injury and/or TNFα-induced phosphorylation in both macrophages and endothelial cells of MEK1/2, a critical component of the ERK pathway which is essential for both injury- and TNFα-induced angiogenesis (Fig. 8). The temporal sequence of TNFα/ORM1 gene expression, with TNFα preceding ORM1 by several hours, is critical for the harmonious unfolding of the angiogenic response. In fact priming of the aorta or isolated endothelial cells with ORM1 essentially abrogates TNFα-induced angiogenesis and significantly reduces the angiogenic response of the aortic wall to injury. Addition of exogenous ORM1 after aortic cultures have been prepared reduces the angiogenic response but cannot completely suppress it because induction of TNFα gene expression and angiogenesis have already been activated during the dissection procedure before ORM1 is added to the culture medium.

Our studies with isolated cells indicate that TNFα is synthesized by macrophages whereas ORM1 is produced by mural cells. ORM1 modulates MEK1/2, p38 and NFkB signaling in macrophages and endothelial cells but not in mural cells. Taken together with previous reports from our laboratory and by others [41], [51], [52], these findings establish a paracrine loop of angiogenic regulation whereby injury-activated macrophages produce TNFα which stimulates production in mural cells of VEGF and ORM1. In this cascade of gene induction, VEGF follows TNFα [41], but precedes ORM1. TNFα stimulates endothelial sprouting both directly [24], [29], [53] and indirectly through VEGF and other TNFα-induced angiogenic regulators. Termination of the TNFα-mediated induction of angiogenesis is mediated by ORM1 through dephosphorylation of MEK1/2 in both macrophages, source of the angiogenic stimuli, and in target endothelial cells. The first several hours of aortic culture during which TNFα gene expression is unchallenged by ORM1 enable the aortic wall to generate sufficient levels of angiogenic stimuli including VEGF to effectively activate the angiogenic cascade. ORM1 contributes to the later stages of the angiogenic response by inducing VEGF production in macrophages (Fig. 8).

Previous studies have demonstrated that ORM1 has the capacity to inhibit TNFα activity [11] and protects mice from TNFα -induced lethality or hepatitis caused by TNFα and galactosamine [12]. Our results indicate that ORM1 can also inhibit TNFα-induced angiogenesis. One possible explanation for these anti-TNFα effects is that ORM1 blocks TNFα by inducing expression of sTNFR1 [11]. This finding, which we have confirmed in our laboratory (unpublished observations), does not however explain the signal transduction selectivity of ORM1 which inhibits TNFα–induced phosphorylation of MEK1/2 and p38 MAPK but not NFkB which is instead hyperphosphorylated. An alternative explanation is that ORM1 alters the relative expression of TNFR1 and TNFR2 and/or differentially influences their activity and downstream signaling pathways [54], [55]. By preserving and even enhancing NFkB-mediated TNFα signals while concurrently inhibiting MEK1/2, ORM1 may facilitate selected responses such as cytokine/chemokine-mediated leukocyte influx while inhibiting unwanted vascular proliferation. The critical importance of the MEK/ERK pathway in angiogenesis is underscored by the observation that the spontaneous and TNFα-induced neovascularization of aortic cultures is markedly inhibited by the MEK1/2 inhibitor PD98059. Thus, high levels of circulating ORM1 in the bloodstream [44] may enable TNFα to promote inflammatory responses without necessarily inducing angiogenesis. Likewise TNFα–induced ORM1 at injury sites may limit the angiogenic response through a negative feedback mechanism of TNFα inhibition which would prevent excessive vascular proliferation. The finding that ORM1 behaves like an inhibitor of TNFα signaling was previously described in a study in which this molecule was shown to suppress excess inflammation in the adipose tissue of obese mice [19]. ORM1 in this report was found to inhibit TNFα-induced phosphorylation not only of p38 and ERK but also of NFκB in mouse 3T3-L1 preadipocytes and RAW264.7 macrophages. Our results confirm the ORM1 effect on TNFα-induced phosphorylation of MAP kinases, but not of NFκB which in our hands was instead unaffected or hyperphosphorylated when rat aortic rings and isolated endothelial cells or macrophages were treated with ORM1 or the ORM1/TNFα combination. These differences may be related to different models systems and/or cell types.

Interestingly ORM1 does not interfere with and actually enhances VEGF-mediated phosphorylation of MAPK and angiogenesis. As a result, ORM1 promotes VEGF-stimulated angiogenesis in the aortic ring model and, after a transient inhibitory effect due to suppression of TNFα activity, it ultimately stimulates the spontaneous angiogenic response of aortic explants over time. ORM1 induces expression in macrophages of VEGF which is a potent stimulator of aortic angiogenesis [40]. Similarly ORM1 dose dependently stimulates angiogenesis in the CAM assay where formation of new vessels is driven by VEGF-mediated mechanisms [56], [57]. In contrast priming with ORM1 reduces the capacity of aortic rings to induce formation of new vessels in a subcutaneous model of angiogenesis where injury and inflammatory stimuli are critical for the angiogenic response [49].

In a previous study Irmak and colleagues reported that ORM1 stimulates endothelial cell migration, supports VEGF-induced endothelial tube formation *in vitro*, and enhances VEGF-induced angiogenesis in the CAM assay [37]. Our studies our consistent with these findings and reconcile the apparently conflicting observations that ORM1 stimulates VEGF-mediated angiogenesis [37] but interferes with the function of TNFα [5], which is also proangiogenic [25], [27]–[29]. The differential effects of ORM1 on the phosphorylation of MEK1/2, a requisite proangiogenic signaling pathway for both TNFα and VEGF, provide an explanation for the inhibitory effect of ORM1 on TNFα– but not VEGF-induced angiogenesis. They also explain the bimodal effect of ORM1 which transiently inhibits the TNFα-driven early stages of the angiogenic response in aortic cultures but promotes the VEGF-mediated later stages of vessel growth. The stimulatory effect of ORM1 on late aortic angiogenesis correlate with increased expression of VEGF as demonstrated by qRT-PCR and ELISA studies.

In summary our study identifies the acute phase reactant ORM1 as an important regulator of the angiogenic response to injury and TNFα stimulation. ORM1 modulates injury-induced angiogenesis in a context- and time-dependent manner by limiting the extent of the initial TNFα-mediated angiogenic response while ensuring that the downstream VEGF-mediated stimulation of the angiogenic process is effectively brought to completion. More studies are however needed to better define the mechanisms by which ORM1 influences TNFα signaling and modulates angiostatic and angiogenic signals during angiogenesis. A better understanding of the ORM1/TNFα system may provide novel insights into the mechanisms that regulate formation of new blood vessels in angiogenesis-dependent pathologies and possibly lead to the identification of new molecular targets for therapeutic intervention.

## Materials and Methods

### Reagents

Purified human ORM1 was obtained from Sigma (St. Louis, MO). Recombinant human ORM1 was generated in our lab as described below. Recombinant human VEGF, rat TNFα, and rat M-CSF were purchased from R&D Systems (Minneapolis, MN). Endothelial basal medium (EBM) was obtained from Lonza (Walkersville, MD). Inhibitors of MEK1/2 (PD98059) and p38 MAPK (SB203580) were purchased from Cell Signaling Technology (Beverly, MA). NFκB inhibitor (CAY10512) was obtained from Cayman (Ann Arbor, MI). Antibodies against phosphorylated or total MEK1/2 (Ser217/221), p38 MAPK (Thr180/Tyr182), and pNFkB/p65 (Ser536) were purchased from Cell signaling Technology. Anti-FLAG antibody was obtained from Sigma. Anti-CD31 antibody for immunoperoxidase studies was from Abcam (Cambridge, MA). Goat HRP-conjugated anti-rabbit and anti-mouse antibodies were purchased from Invitrogen (Carlsbad, CA). Protease and phosphatase inhibitor cocktail and chemiluminesence reagents were from Pierce (Rockford, IL).

### *Ex vivo* Aortic Ring Assay

All animal procedures were performed in accordance with Veterans Administration Puget Sound Health Care System institutional animal care and use committee and NIH guidelines. Rat aortic rings were prepared, embedded in collagen gels, and cultured in serum-free EBM as reported [58]. Cultures were supplemented with TNFα (5 ng/ml), ORM1 (1–100 µg/ml), VEGF (10 ng/ml), TNFα/ORM1, TNFα/VEGF, or left untreated. To evaluate the role of signal transduction pathways in aortic angiogenesis, cultures were also treated with inhibitors of MEK1/2 (PD98059), p38 MAPK (SB203580), or NFκB (CAY50512) signaling. Growth medium with or without added reagents was changed three times a week starting from day 3. In a separate set of experiments aortic tubes were incubated overnight in serum-free EBM containing ORM1 and cross-sectioned in ORM1-containing medium to obtain rings. The aortic rings were then rinsed and cultured in serum-free EBM with or without TNFα or VEGF. The angiogenic response of aortic cultures was measured by counting the number of neovessels over time, as reported [39].

### Cell Isolation

Rat aortic endothelial and mural cells were isolated from the rat aorta as described [59], [60]. Rat aortic macrophages (RAM) were isolated from M-CSF-treated aortic cultures as reported [50], [61]. Rat bone marrow derived macrophages (RBMM) were isolated according to standard techniques [41], [50], [61]. Different cell types were characterized with specific markers of cell differentiation [40], [41], [50], [59]–[61].

### Endothelial Collagen Invasion Assay

Wells of a 4-well dish (NUNC) were filled with 300 µl of rat tail collagen which was allowed to polymerize for 15 minutes at 37°C. After polymerization each gel was seeded with 200,000 rat aortic endothelial cells in 0.5 ml of EBM medium containing 10% fetal bovine serum. The next day the cultures were rinsed with EBM, incubated for 6 hours in EBM with or without 100 µg/ml ORM1, rinsed and then treated with: (A) bFGF and VEGF; (B) bFGF, VEGF, and TNFα; (C) or left untreated. All invasion assays were carried out in serum-free EBM. bFGF and VEGF were used at 50 ng/ml concentration and TNFα at 10 ng/ml. In a separate set of experiments the endothelial invasion assay was performed on collagen gels containing 200,000 rat bone marrow macrophages. Endothelial cultures with or without macrophages were primed with ORM1 as described above and then treated with the bFGF/VEGF combination or left untreated. After 48–72 hours cultures were fixed in 4% paraforlmaldehyde and stained with 1% Methylene blue/1% Azure II (Sigma) in 1% sodium borate. Endothelial sprouting was measured by image analysis using ImageJ NIH software. Fields with the highest degree of endothelial sprouting in the central regions of the culture wells were photographed at 10× magnification. Six fields per experimental condition were selected for image analysis. Each image was digitally thresholded to highlight endothelial sprouts and exclude endothelial monolayers and isolated macrophages.

### *In vivo* Aortic Ring Assay

Constructs were prepared and implanted in syngeneic rats as reported [49]. Each construct consisted of an aortic ring-containing collagen gel (7 mm) supported by Gelfoam (11 mm). The day before implantation aortic rings were kept in serum-free EBM with or without 100 µg/ml ORM1 at 37°C in a humidified CO_2_ incubator. The next day aortic rings were incorporated into the gel constructs and kept in serum-free medium with or without ORM1. Recipient animals were anesthetized with 2–4% isoflurane in oxygen. After making bilateral incisions in the animal's dorsum and creating pouches by blunt dissection, 2 constructs per animal were inserted with a spatula into the subcutaneous space. Animals were maintained for 9 days prior to sacrifice and removal of implants for immunohistochemical and image analysis studies. Angiogenesis was measured by performing image analysis on frozen sections stained by immunoperoxidase for the endothelial marker CD31 [49]. Images were thresholded and quantitatively evaluated with ImageJ NIH software as described above.

### Chorioallantoic Membrane Assay

The CAM assay was performed on chick embryos grown in shell-less cultures [62]. Briefly, fertilized eggs of White Leghorn chickens (Sunnyside, Beaver Dam, WI) were kept for 3 days at 37.5°C in a humidified incubator, dipped in 70% ethanol, dried and opened with a razor blade. The egg contents were poured into clear plastic wrap hammocks suspended in sterile plastic holders with tripods and covered with the lid of a Petri dish [62]. After 7 days, discs made from 20 µl drops of 0.5% methylcellulose containing 0, 50, 100, 200, or 300 ng of ORM1 were placed onto the CAM (10-day-old); nuclease free water was used as negative control. Each ORM1 dose was tested on four embryos. After placement of the discs, embryos were transferred back into the incubator. After 3 days, embryos were examined under a stereomicroscope for evaluation of angiogenesis. To improve vessel resolution, pre-warmed whipping cream was injected under the CAM. Angiogenesis was quantitated by counting vessel intersections.

### Western Analysis

Aortic protein extracts for signaling studies were obtained as described [63]. Briefly, isolated pieces of rat aorta were snap frozen in liquid nitrogen and pulverized before being transferred to RIPA buffer (Pierce). Extracts from cells were harvested directly into RIPA buffer. All samples were boiled for 5 minutes in Laemmli buffer (60 mM Tris-Cl pH 6.8, 2% SDS, 10% glycerol, 5% β-mercaptoethanol, 0.01% bromophenol blue) and run in a 4–15% polyacrydamide gradient gel (Bio-Rad, Hercules, CA) under denaturing conditions. Proteins were then transferred to a PVDF membrane (Pierce), blocked with blocking buffer (Pierce) and probed with phosphospecific antibodies. The blots were then stripped and reprobed with antibodies against total proteins. Specific antibody binding was detected with the ECL system (Pierce).

### ELISA

ELISA was used to measure levels of VEGF (R&D Systems) and ORM1 (Genway Biotech, San Diego, CA) in the conditioned medium of aortic cultures.

### Cloning of Human ORM1

Human ORM1 cDNA was generated by reverse transcribing total RNA isolated from the EAhy926 endothelial cell line (American Type Culture Collection, Manassas, VA) treated with TNFα for 6 hours. This was used as a template for PCR to make the full length ORM1 cDNA. The following oligonucleotides were used for the PCR reaction: 5′ ATAAGCTTATGGCGCTGTCCTGGGTTCTTACA 3′ (forward) and 5′ ATGGATCCTTACTTGTCATCGTCATCCTTGTAATCGGATTCCCCCTCCTCCTGTTT 3′ (reverse). The amplicon was digested with HindIII and BamHI and cloned into the pcDNA3.1 vector (Invitrogen) resulting in a full length ORM1 cDNA containing a FLAG-TAG at the 3′ end. The construct was sequenced on both strands to verify accuracy of amplicon cloning.

EAhy926 cells were transfected with pcDNA3.1/ORM1cDNA or an empty vector using the FuGene6 reagent (Roche Diagnostics, Indianapolis, IN). Transfected cells were selected in the presence of 600 µg/ml G418 (Sigma) for seven to ten days and single clones were isolated with sterile cotton swabs (Ted Pella Inc, Redding, CA). Clones were transferred into a 24-well plate and amplified by sequential transfers into larger dishes. Serum-free medium conditioned for 48 hours by single or pooled clones was analyzed for the presence of ORM1 by Western blotting using anti-Flag antibody (Sigma).

### Standard and Quantitative Real-Time PCR (qRT-PCR)

Total RNA was extracted from aortic rings embedded in collagen gels or from aortic macrophages, endothelial cells, or mural cells using the RNAEasy Micro kit (Qiagen, Valencia, CA). cDNA was synthesized by reverse transcription (RT-PCR) using 100 ng of total RNA in random-primed reverse transcription reactions using Superscript III (Invitrogen). Reactions lacking enzyme were used as negative controls. PCR reactions were carried with 1/20^th^ of the cDNA and the following primers: ORM1: 5′-GTGTGCAGGAGCAGTGAAAA-3′ and 5′-CATGCCCACATCTTTGACAG-3′; GAPDH: 5′- GGTGGACCTCATGGCCTACA-3′ and 5′-TGGGTGGTCCAGGGTTTCT-3′. Briefly, GoTaq polymerase and GoTaq reagents (Promega) were used to generate amplicons from cDNA using the following conditions, 1 min 94 C, 1 min 55 C, and 1 min 72 C for 30 cycles. Resulting amplicons were separated by gel electrophoresis on 2% agarose gels and visualized by UV light after staining with ethidium bromide.

Quantitative reverse transcription polymerase chain reactions (qRT-PCR) were carried out to examine the expression of TNFα, VEGF and ORM1. In each case reactions were set up using 1/20 of the RT reaction as template with SYBR-green reagents (Applied Biosystems, Foster City, CA) and the following gene specific primers (Invitrogen): TNFα: 5′-TCGGGGTGATCGGTCCCAACAA-3′ and 5′-GCTACGGGCTT GTCACTCGAGTT-3′; VEGF-A: 5-GGGAGCAGAAAGCCCATGAAGTG-3 and 5′-CCAGGGTCTCAATTGGACGGCAAT-3′; ORM1: 5′-GTGTGCAGGAGCAGTGAAAA-3′ and 5′-CATGCCCACATCTTTGACAG-3′; HIF1α: 5′-CCGGCGGCGAGAACGAGAAGAAA-3′ and 5′-TCTTTGCTTCGCCGAGATCGTGC-3′; β-Actin: 5′-GGGAAATCGTGCGTGACATT-3′ and 5′-GCGGCAGTGGCCATCTC-3′. All reactions were carried out in triplicate and expression measured with a ABI 7500 thermal cycler and Prizm software. The 2-ΔΔCt method was used to calculate ratios of gene expression normalized to β-Actin in the same sample [64].

### Statistical Analysis

Student's T-test was used for statistical analysis of *in vitro*, *ex vivo* and *in vivo* experiments. Statistical significance was set at p<0.05.
